# Open Latarjet Procedure for Anterior Shoulder Instability: A Correlation Between Preoperative Imagistic Landmarks and 6-Month Follow-Up

**DOI:** 10.3390/jcm15218230

**Published:** 2026-10-22

**Authors:** Arpad Solyom, Denes Marton, Flaviu Moldovan, Fodor Pal, Evelyn Kovács, Zoltán Derzsi, Florin Stoica-Buracinschi, János Székely, Andrei Căliman

**Affiliations:** 1 Department of Orthopedics—Traumatology, “George Emil Palade” University of Medicine, Pharmacy, Science, and Technology of Târgu Mureș, 540142 Targu Mures, Romania; arpad.solyom@umfst.ro (A.S.); flaviu.moldovan@umfst.ro (F.M.); pal.fodor@umfst.ro (F.P.); 2 Târgu Mureș County Clinical Emergency Hospital, 540136 Targu Mures, Romania; drmartond@gmail.com (D.M.); kovacs.evelyn.24@stud.umfst.ro (E.K.); 3 Discipline of Pediatric Surgery and Orthopedics, Faculty of Medicine, “George Emil Palade” University of Medicine, Pharmacy, Science, and Technology of Târgu Mureș, 540142 Targu Mures, Romania; zoltan.derzsi@umfst.ro; 4 Department of Medical Informatics and Biostatistics, Faculty of Medicine, “George Emil Palade” University of Medicine, Pharmacy, Science, and Technology of Târgu Mureș, 540142 Targu Mures, Romania; florin.stoica@umfst.ro; 5 IOSUD Doctoral School, “George Emil Palade” University of Medicine, Pharmacy, Science, and Technology of Târgu Mureș, 540142 Targu Mures, Romania; egyjanek@yahoo.com

**Keywords:** open Latarjet procedure, Hill–Sachs interval, glenoid track, glenoid index, glenoid bone loss, auto-Rowe score, auto-Walch–Duplay score

## Abstract

**Background:** The open Latarjet procedure is widely used for the treatment of recurrent anterior shoulder instability associated with bipolar bone defects. Preoperative imaging parameters may be associated with short-term functional outcomes following the open Latarjet procedure for recurrent anterior shoulder instability. **Materials and Methods:** This retrospective study included 16 patients treated with the modified open Latarjet procedure. Preoperative CT or MRI measurements included glenoid bone loss, Hill–Sachs interval (HSI), glenoid track (GT), HSI/GT ratio and glenoid index. At 6 months, patients were evaluated using the auto-Rowe and auto-Walch–Duplay scores, range of motion, recovery time, residual symptoms, and return to activity. **Results:** The median Rowe and Walch–Duplay scores were 95 (90–95) and 75 (70–90), respectively. Fifteen patients resumed physical activity, including seven who returned to their previous level. No procedure-related complications were reported. Among the imaging parameters analysed, a higher HSI/GT ratio was associated with lower Walch–Duplay (*p* = 0.007, q = 0.043) and Rowe scores (*p* = 0.014, q = 0.043). Higher glenoid bone loss was associated with a lower Rowe score (*p* = 0.015, q = 0.043), while a higher glenoid index was associated with a lower Walch–Duplay score (*p* = 0.017, q = 0.043). Glenoid track showed a positive nominal association with the Walch–Duplay score (*p* = 0.036), which did not remain significant after FDR adjustment (q = 0.072). **Discussion:** Preoperative assessment of bipolar bone loss, particularly the HSI/GT ratio, may provide complementary information for surgical planning and for exploring associations with short-term functional outcomes. The formula proposed by Boden may help determine whether the coracoid graft is sufficient to convert an off-track lesion into an on-track lesion. In patients with larger Hill–Sachs defects, additional remplissage or humeral head bone grafting may be considered, although these techniques carry risks of external rotation restriction and graft resorption or osteonecrosis, respectively. **Conclusions:** The open Latarjet procedure is a widely used surgical option for anterior shoulder instability. These exploratory findings require validation in larger cohorts with longer follow-up.

## 1. Introduction

Through dynamic stability provided by the rotator cuff and static stability supplied by the articular capsule and glenohumeral ligaments, the shoulder has one of the most complex ranges of motion. This inherent anatomical mobility predisposes the glenohumeral joint to instability, especially in young athletic patients [1,2]. The typical injury requires abduction combined with external rotation, which generates a force directed from posterior to anterior [3,4]. This mechanism explains the higher rate of anterior dislocation (80–97%) than posterior dislocation or luxatio erecta [5,6,7].

Recurrent anterior shoulder instability is a multifactorial condition that may involve several associated structural abnormalities, including Bankart and Hill–Sachs lesions, glenoid bone loss or rotator cuff tears [8,9]. According to multiple studies, the open Latarjet procedure (transferring the coracoid process and its attached conjoint tendon to the anterior glenoid rim) is one of the most successful surgeries, with excellent clinical outcomes in the short term, high rates of return to sport and low recurrence rates at medium-term follow-up, particularly in patients with a minimum of 15% glenoid bone loss [10,11].

The present study was designed to assess the relationship between preoperative imaging characteristics and short-term functional outcomes at 6 months following the modified open Latarjet procedure. Taking into account the lesions identified on magnetic resonance or computed tomography scans and the short-term outcomes, the surgeon can improve intraoperative management and, eventually, the quality of medical performance.

## 2. Materials and Methods

A retrospective study was conducted between January 2021 and June 2026 following approval by the institutional ethics committee and in accordance with the principles of the Declaration of Helsinki. In total, 17 consecutive patients were treated in our hospital for recurrent anterior shoulder instability with an open Latarjet procedure, according to multiple studies’ indications (Table 1) [10].

### 2.1. Eligibility Criteria

Of these patients, 16 met inclusion criteria: a. traumatic history, b. more than two episodes of dislocation, c. no rotator cuff pathology, d. recurrent anterior dislocation, e. no previous shoulder surgery, f. a minimum follow-up of 6 months, and g. complete evaluation (preoperative, perioperative and postoperative) (Figure 1).

### 2.2. Preoperative Evaluation

During the clinical examination, all the patients presented a positive triple test (apprehension, relocation and release tests). All imaging measurements were independently performed by two physicians, and any discrepancies or uncertainties between their assessments were resolved by a third observer through consensus review. Shoulder magnetic resonance imaging (MRI) was used to identify associated lesions, including rotator cuff tears, labral injuries, and Hill–Sachs lesions. MRI was also used to determine the Hill–Sachs interval (Figure 2), defined as the distance between the rotator cuff insertion and the medial margin of the Hill–Sachs lesion. Computed tomography (CT) scans were used to assess glenoid morphology and to quantify glenoid bone loss, as well as to calculate the glenoid index, glenoid track (Figure 3), and glenoid version, after a 3D Multiplanar Reconstruction (Figure 4) (Table 2).

### 2.3. Intraoperative Aspects

The Modified Latarjet procedure was performed using a deltopectoral approach, with the patient in a beach-chair position. The coracoid process (CP) was identified. After the dissection of the coracoacromial ligament and the pectoralis minor tendon, the osteotomy of the CP was accomplished, with the conjoint tendon attached (a strong band of tissue formed by the short head of the biceps brachii and the coracobrachialis muscles). Considering the findings of multiple studies reporting improved postoperative internal rotation range of motion [12,13], the subscapularis muscle was split between the inferior and middle thirds, and the capsulectomy was performed parallel to the joint line. The anterior rim of the glenoid (between 3 o’clock and 6 o’clock) and the bone graft were decorticated, and the osteosynthesis was achieved with 2 cancellous screws placed approximately 10 mm apart.

After the osteosynthesis (Figure 5), only the subcuticular tissue and the skin were closed. Capsular repair was not performed, as previous studies have suggested that it may be associated with reduced postoperative external rotation, although the available evidence remains limited [14,15,16].

### 2.4. Postoperative Care and Evaluation

Following surgery, the affected limb was immobilised in a neutral-position shoulder brace for 4–6 weeks. After 3 weeks, the rehabilitation program was initiated and progressed through three sequential phases (Table 3) [17]. At the 6-month follow-up, all 16 patients underwent clinical evaluation using the validated self-administered version of the Walch–Duplay and Rowe score (auto-Walch–Duplay, auto-Rowe), following the model proposed by Lazrek et al. [18].

### 2.5. Statistical Evaluation

A database for hospital records was developed using Microsoft Excel. Statistical analyses were performed using R statistical software (R Foundation for Statistical Computing, Vienna, Austria [19]). Continuous and categorical variables were summarised using appropriate descriptive statistics, with 95% confidence intervals. No missing data were present for imaging or clinical outcomes. Parametric continuous variables were reported as mean ± standard deviation (SD), whereas non-parametric continuous variables were presented as median and interquartile range (IQR); categorical variables were summarised as n (%). Because of the small sample size and non-Gaussian data distributions, nonparametric statistical methods were primarily used. Associations between imaging parameters and clinical outcomes, as well as between the number of previous dislocations and glenoid bone loss or the HSI/GT ratio, were assessed using Spearman correlations, 95% confidence intervals, and *p*-values. Return-to-activity groups were compared using exact Mann–Whitney tests or sample t-tests, depending on data distribution. Statistical interpretation focused on q-values, *p*-values, confidence intervals and false-discovery rate, using the Benjamini–Hochberg procedure. The 95% confidence intervals were obtained using 10,000 bootstrap resamples with replacement. Nominal statistical significance was defined as *p* < 0.05, whereas FDR-adjusted significance was defined as q < 0.05. Reporting was structured according to STROBE principles [20].

Given the limited sample size, all analyses were considered exploratory and should be interpreted with caution, as the study was not powered to establish definitive prognostic associations or treatment effectiveness.

## 3. Results

### 3.1. Characteristics of the Study Sample

Sixteen patients were included, of whom 12 were men (75.0%). The median age was 25 (IQR: 20–42.5), and 9 patients were younger than 30 years. The right shoulder was operated on in 9 patients, and the dominant shoulder in 7. The median number of preoperative dislocations was 9.5 (IQR: 7.5–15.0). The preoperative imaging landmarks were calculated using the formulas presented in Table 2. Within the analysed cohort, glenoid anteversion was identified in two patients (Table 4).

### 3.2. Functional Outcomes and Return to Activity

Median recovery time was 4.0 months (IQR: 3.0–6.0). Median Rowe score was 95 (IQR: 90.0–95.0), and median Walch–Duplay score was 75 (IQR: 70–90). Seven patients (43.8%) returned to the same level. A total of 15 patients (93.8%) continued some form of activity, including the previous level, a lower level, or a different sport. No procedure-related complications were reported in any of the patients (Table 5).

### 3.3. Radiologic–Clinical Associations

#### 3.3.1. HSI/GT Ratio

The Hill–Sachs interval-to-glenoid track width ratio (1.41 ± 0.23) is an important parameter for evaluating recurrent anterior shoulder instability because it integrates humeral and glenoid bone defects into a single measurement. Among the variables analysed, the HSI/GT ratio showed the strongest association with postoperative outcomes. A higher ratio was associated with a lower Walch–Duplay score (*p* = 0.007) (Figure 6A) and a lower Rowe score (*p* = 0.014) (Figure 6B) (Table 6).

#### 3.3.2. Glenoid Index (GI)

The glenoid index appears to be a useful supplementary marker for identifying patients at increased risk of anterior shoulder instability. The mean glenoid index was 1.77 ± 0.18 and showed an inverse association with Walch–Duplay (*p* = 0.017) (Figure 7A) (Table 6).

#### 3.3.3. Glenoid Bone Loss

Glenoid bone loss was another imaging parameter associated with less favourable functional outcomes, with a mean value of 22.19 ± 5.17% in the study. GBL showed an inverse association with the Rowe score (*p* = 0.015) (Figure 7B). Its relationship with the Walch–Duplay score was negative and non-significant (Table 6).

#### 3.3.4. Glenoid Track

The glenoid track (2.00 ± 0.22), representing the contact zone between the glenoid articular surface and the humeral head, was associated with higher Walch–Duplay scores at the nominal level (*p* = 0.036) (Figure 7C) (Table 6).

#### 3.3.5. Hill–Sachs Interval

The median Hill–Sachs interval was 2.77 (IQR: 2.64–2.83). Higher Hill–Sachs interval values tended to be associated with lower Rowe and Walch–Duplay scores, although these findings were not statistically significant. (Table 6).

A large Hill–Sachs interval is associated with an increased risk that a Latarjet will fail to convert an off-track lesion to on-track, but HSI alone is not an independently validated universal predictor of Latarjet failure [21].

### 3.4. Postoperative Activity Level

To evaluate outcomes by return to activity, the study cohort was divided into two groups: patients who returned to the same level of activity (same level) and those who did not regain their preoperative activity level (other outcome) (Figure 8). Comparison between the two groups showed numerical differences in external rotation and Rowe scores; however, none of these differences reached statistical significance. Patients who returned to the same level had a lower mean HSI/GT ratio than the other patients (1.27 ± 0.13 vs. 1.52 ± 0.23) (*p* = 0.021). Glenoid bone loss and glenoid index were numerically lower in the same-level group, but these comparisons were not nominally significant (Table 7).

The Walch–Duplay score was not treated as an independent validation because it contains an activity/sport domain, and therefore, a higher outcome was expected for those who returned at the same level.

### 3.5. Association Between the Number of Previous Dislocations and Bipolar Bone Loss

Exploratory analyses showed no significant association between the number of preoperative dislocations and glenoid bone loss (Spearman’s ρ = −0.02, *p* = 0.930) or the HSI/GT ratio (ρ = 0.15, *p* = 0.570).

### 3.6. False-Discovery-Rate-Adjusted Results

After Benjamini–Hochberg false-discovery-rate adjustment within the radiologic–clinical testing family (Table 6), the associations of the HSI/GT ratio with the Walch–Duplay and Rowe scores, glenoid bone loss with the Rowe score, and glenoid index with the Walch–Duplay score remained statistically significant (all q = 0.043). The association between glenoid track and the Walch–Duplay score did not remain significant after FDR adjustment (q = 0.072). In the separate family of exploratory between-group comparisons reported in Table 7, none of the associations remained statistically significant after FDR correction. Given the small sample size and exploratory design, these findings should be interpreted cautiously and require confirmation in larger, independent cohorts.

## 4. Discussion

The open Latarjet procedure may be considered an effective surgical option for recurrent anterior shoulder instability, particularly in patients with significant glenoid bone loss and off-track Hill–Sachs lesions. By increasing the effective glenoid articular arc through coracoid transfer, the procedure can restore the glenoid track and convert an off-track lesion into an on-track configuration, thereby reducing the risk of recurrent engagement and instability. In this context, preoperative imaging represents an important component of surgical planning. Within the present exploratory cohort, the HSI/GT ratio, glenoid index, and glenoid bone loss demonstrated associations with selected short-term functional outcomes that remained below the FDR-adjusted significance threshold within the radiologic–clinical testing family. The glenoid track demonstrated a nominal association with the Walch–Duplay score but did not remain significant after FDR adjustment. These findings should be interpreted as exploratory associations rather than evidence of predictive performance or recurrence-risk stratification. Furthermore, these imaging parameters should not be regarded as completely independent effects because the glenoid track is directly influenced by glenoid bone loss, while the HSI/GT ratio incorporates the glenoid track in its denominator.

The observed association between a less favourable preoperative osseous configuration and lower functional scores may reflect multiple clinical factors, including residual apprehension, pain, restricted range of motion, and limitations in activities of daily living or sports participation. Nevertheless, the individual domains of the Rowe and Walch–Duplay scores were not analysed separately; therefore, the relative contribution of each component to the overall score cannot be established. This is particularly relevant, especially for the Walch–Duplay score, which includes an activity/sport component and may consequently be partly influenced by the extent of return to previous physical activity.

Although most patients resumed some form of physical activity, fewer than half returned to their previous activity level. Patients who returned to the same level had a lower HSI/GT ratio, suggesting a possible association between the severity of bipolar bone loss and postoperative activity recovery. However, this finding should be interpreted cautiously, as detailed information regarding sport type, competitive level, and preoperative activity level was not systematically available.

Recurrent episodes of instability may contribute to the progression of bipolar bone loss; however, in the present cohort, the number of preoperative dislocations was not significantly associated with either glenoid bone loss or the HSI/GT ratio.

The relatively short follow-up period represents an important limitation of the present study. The 6-month assessment provides information regarding early functional recovery and return to physical activity; however, it does not allow reliable evaluation of medium- or long-term outcomes following the Latarjet procedure. In particular, recurrent instability, delayed complications, graft remodelling or resorption, degenerative glenohumeral changes, and the durability of return to sport may become evident only with longer follow-up. Therefore, the present findings should be interpreted as short-term exploratory observations and should not be extrapolated to long-term clinical effectiveness or durability of the procedure.

To improve the understanding of preoperative planning, Boden et al. [22] proposed determining the coracoid process width and comparing it to the subtraction of the GT from the HSI (ΔHSI-GT). A coracoid width exceeding the calculated value was considered sufficient to convert the lesion from off-track to on-track. The predicted final glenoid width was determined by subtracting the extent of bone loss from the native glenoid diameter and subsequently adding the coracoid graft width. Following the assessment of these measurements, statistically significant improvements in postoperative outcomes were observed.

Preoperative bone-loss parameters represent only one component of surgical outcome. Postoperative factors such as coracoid graft position, graft union, remodelling, and resorption may also modify the final glenoid geometry and therefore influence stability. These variables were not systematically quantified in the present study and should be incorporated into future radiologic–clinical analyses.

Although a higher HSI/GT ratio was associated with less favourable functional outcomes in the present cohort, this association should not be interpreted as supporting the routine use of an additional humeral-sided procedure. Additional procedures, such as remplissage or humeral head bone grafting, should therefore be considered on a selective basis, particularly in cases in which the coracoid graft is unlikely to provide sufficient restoration of the glenoid track or when residual engagement is anticipated [23]. Abd-Elzaher et al. [24] reported significant postoperative improvements for patients with recurrent anterior shoulder dislocation with glenoid bone loss >25% and off-track lesions, in both the isolated Latarjet and the Latarjet combined with remplissage groups. However, the remplissage group exhibited a significant restriction in external rotation.

When anatomical restoration of the humeral head articular arc is required, reconstruction of the Hill–Sachs defect using bone grafting represents an anatomical strategy aimed at restoring humeral head sphericity and joint congruence. Restoration of articular congruity increases the glenohumeral contact area and promotes a more uniform distribution of joint contact stresses [25,26,27]. Moreover, correction of the engaging Hill–Sachs lesion may facilitate improvement in postoperative range of motion, except for external rotation. Despite the observed gain in postoperative range of motion, Liow et al. [28] and Saltzman et al. [29] reported a higher rate of allograft necrosis and resorption.

## 5. Conclusions

In this retrospective series of 16 patients treated with the modified open Latarjet procedure, several preoperative imaging parameters showed nominal associations with short-term functional outcomes at 6 months. The HSI/GT ratio showed inverse associations with the Walch–Duplay and Rowe scores, while glenoid bone loss and glenoid index were associated with selected functional outcomes. These associations remained below the FDR-adjusted significance threshold within the radiologic–clinical testing family. The glenoid track showed a nominal association with the Walch–Duplay score that did not remain significant after FDR adjustment.

These findings should therefore be considered exploratory and hypothesis-generating. Longer-term prospective studies with larger cohorts and standardised postoperative clinical and imaging follow-up are required to determine whether these preoperative imaging parameters have prognostic value for recurrence, sustained functional recovery, graft-related outcomes, and long-term return to activity.

## 6. Limitations

- Small sample (n = 16), with wide confidence intervals.
- Short follow-up duration of 6 months, which limits the assessment of recurrent instability, long-term functional outcomes, graft-related changes, degenerative changes, and sustained return to sport.
- Retrospective design and self-report outcomes questionnaire.
- No sport type, competitive level, return criteria, or psychological scale such as SIRSI.
- All patients had HSI/GT >1; there was no on-track comparison group.
- The absence of systematic postoperative CT assessment.

## Figures and Tables

**Figure 1:**
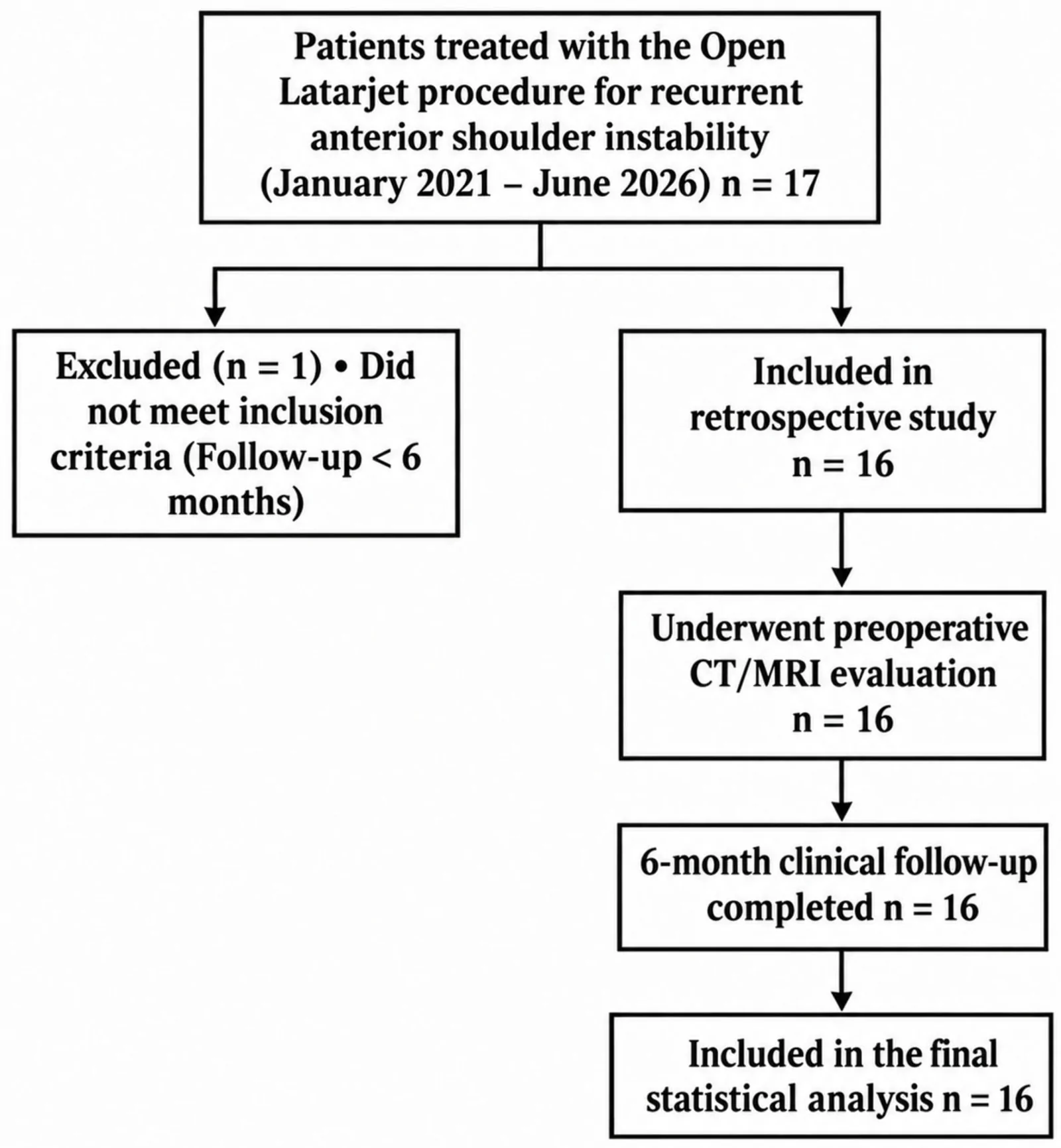
Flowchart of patient selection and inclusion in the study.

**Figure 2:**
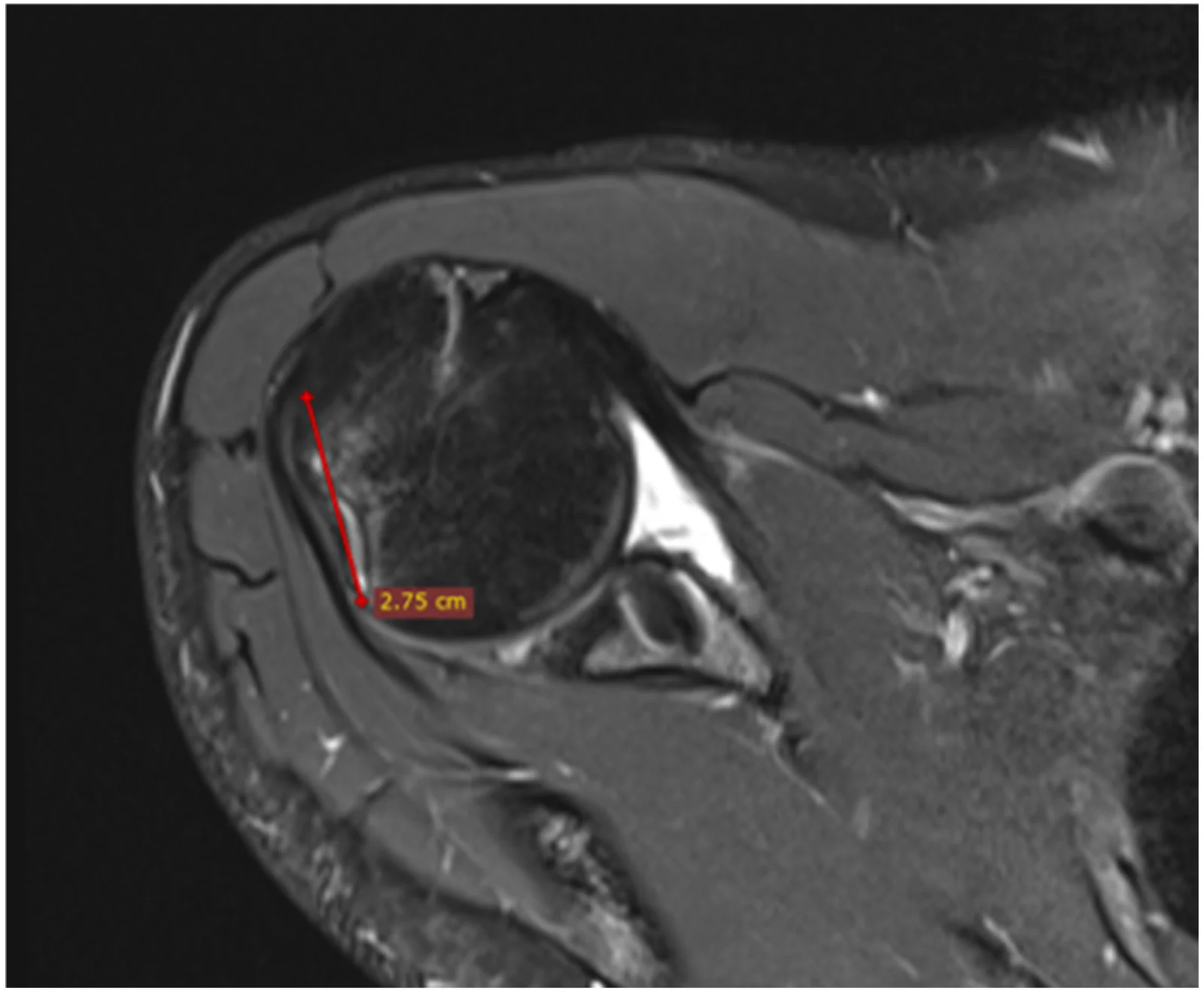
HSI—Right shoulder MRI (PD_TSE_FS, axial).

**Figure 3:**
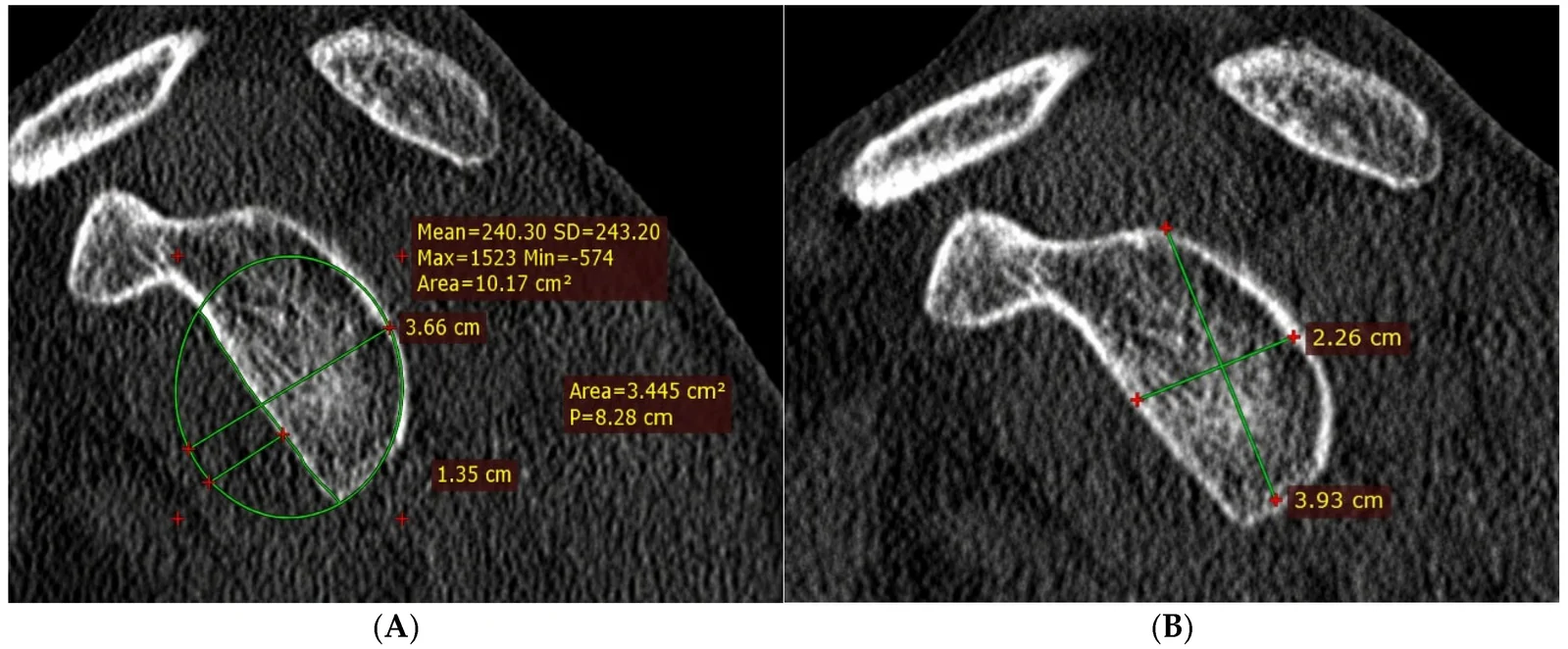
(**A**) GI—Left shoulder CT scan (sagittal view); (**B**) GBL + GT—Left shoulder CT scan (sagittal view).

**Figure 4:**
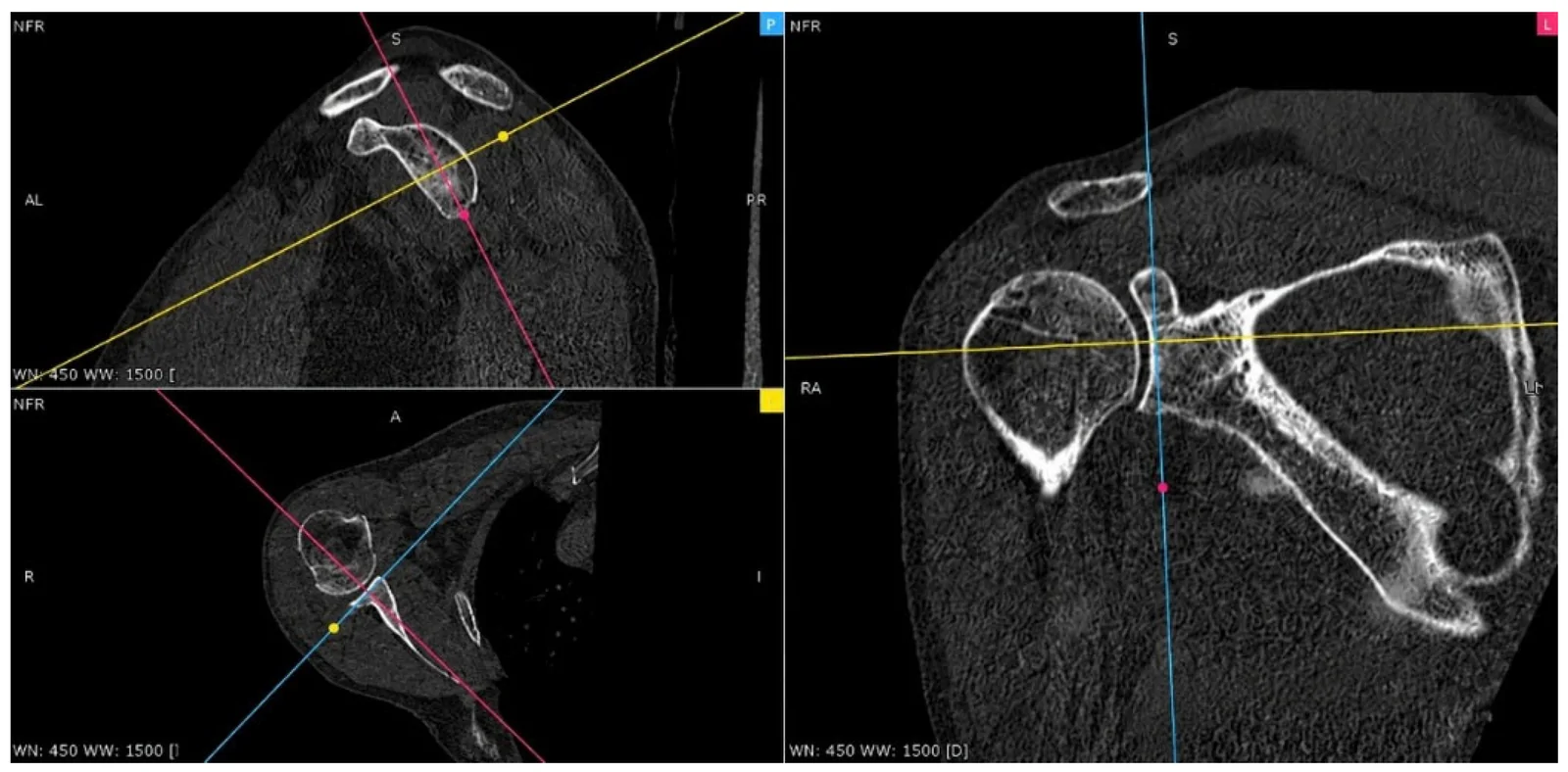
CT—3D multiplanar reconstruction.

**Figure 5:**
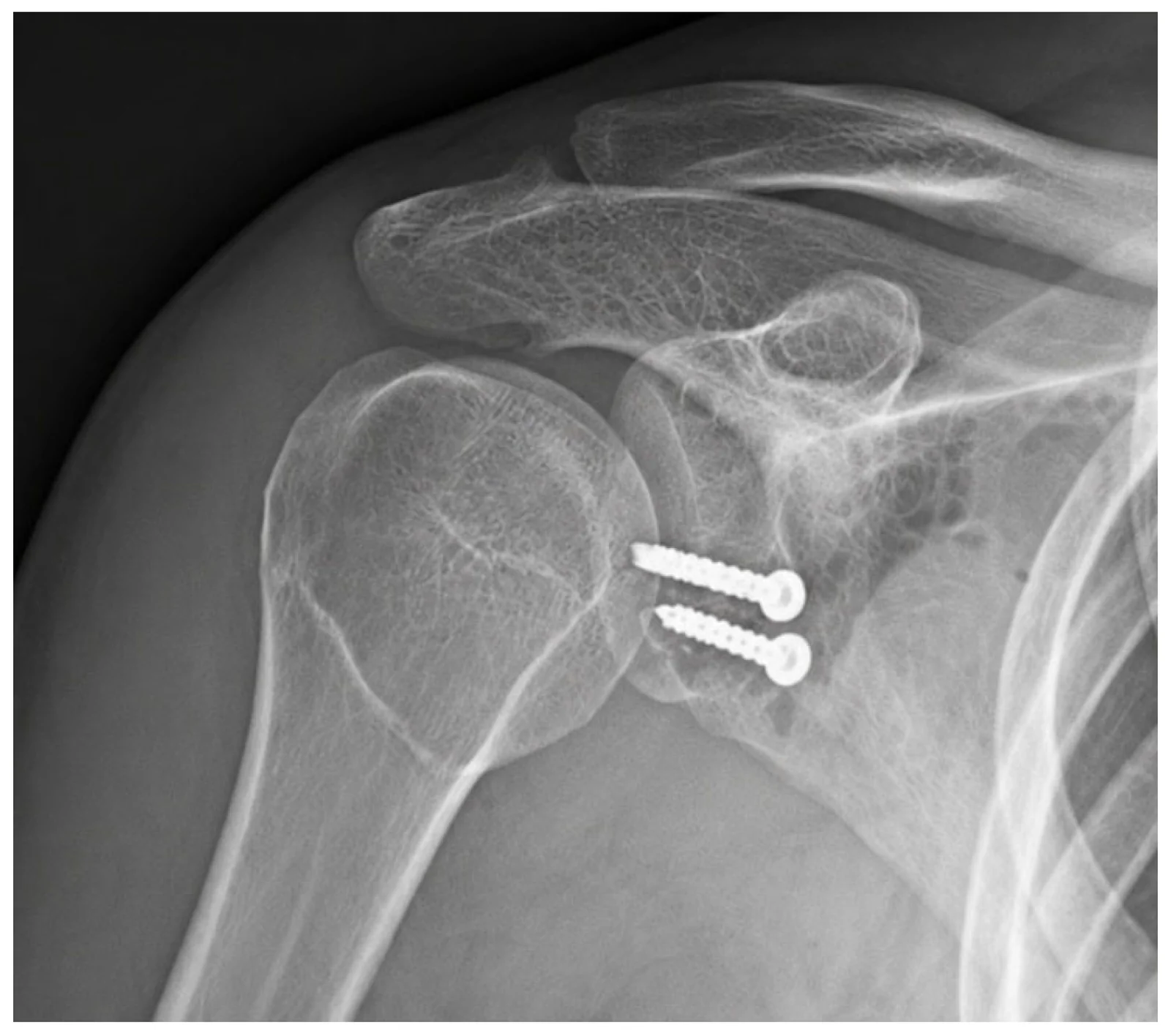
Postoperative shoulder X-ray.

**Figure 6:**
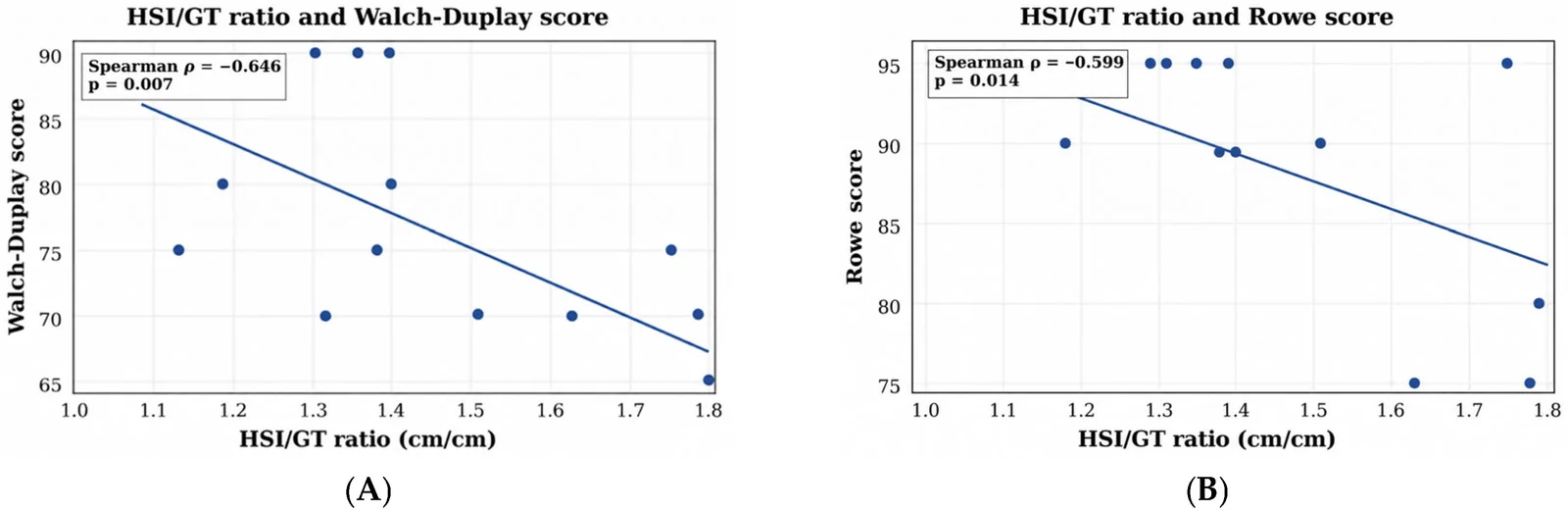
(**A**) Correlation between HSI/GT ratio and Walch–Duplay score; (**B**) correlation between HSI/GT ratio and Rowe score.

**Figure 7:**
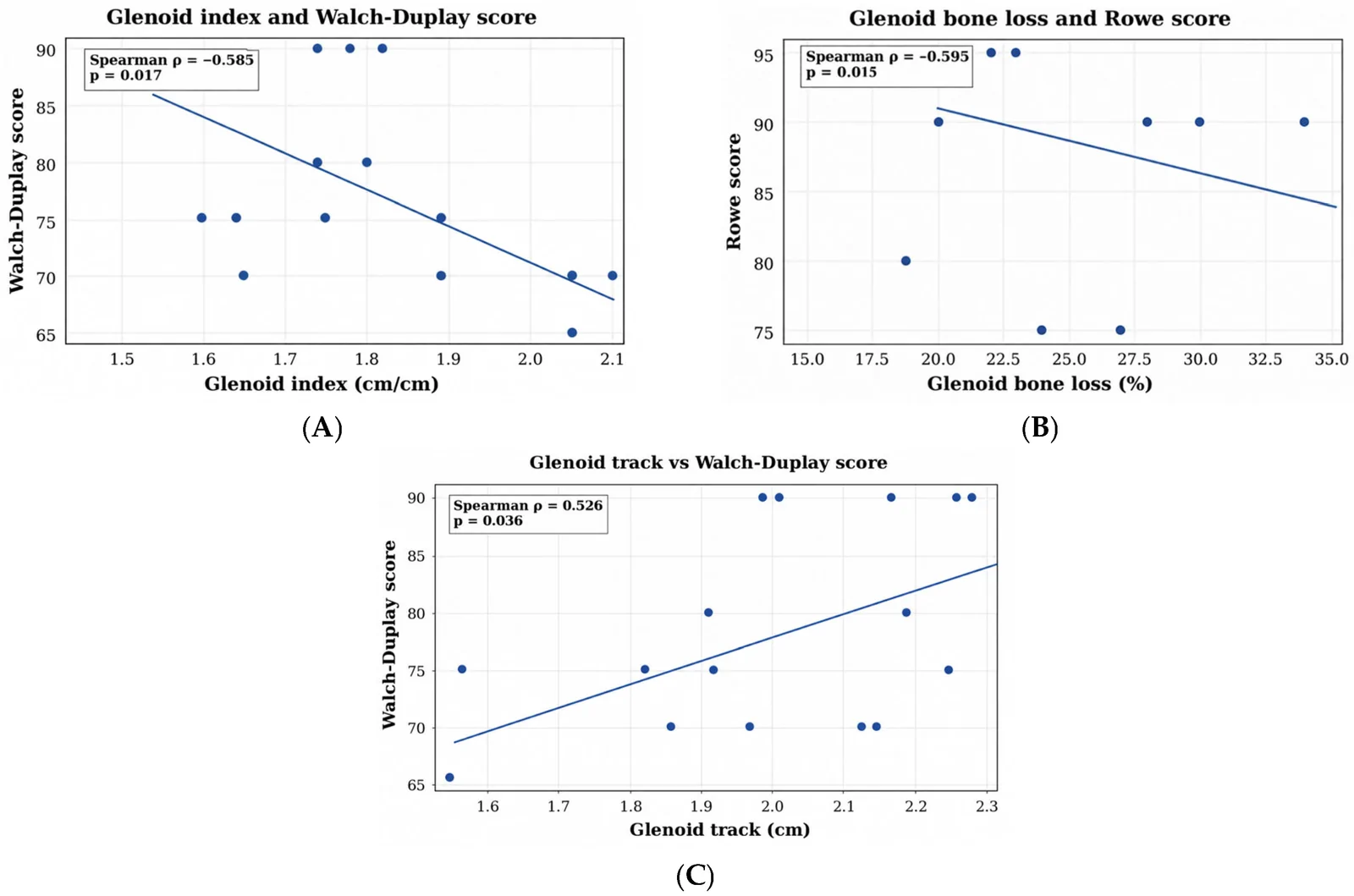
(**A**) Correlation between GI and Walch–Duplay score; (**B**) correlation between GBL and Rowe score; (**C**) correlation between GT and Walch–Duplay score.

**Figure 8:**
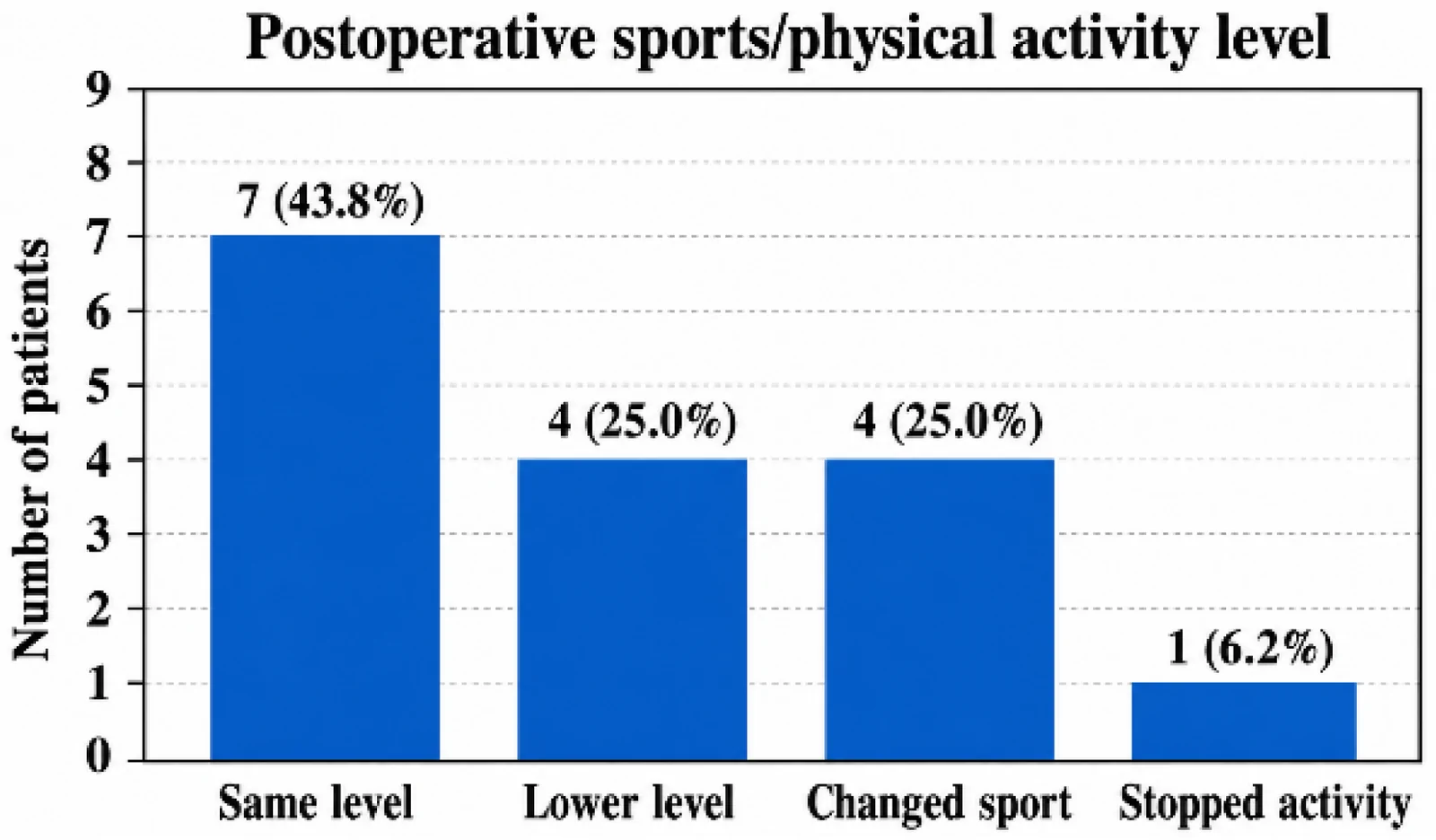
Distribution of postoperative sports/physical activity level.

**Table 1:** Relative indications and contraindications for the Modified Latarjet procedure.

Relative Indications for Open Latarjet Procedure	Relative Contraindications for Open Latarjet Procedure
Recurrent instability	Multidirectional instability
Failed prior surgery	Voluntary dislocation
Collision athlete	Uncontrolled epilepsy/seizure disorders
Critical glenoid bone-loss (15–20%)	Irreparable rotator cuff tear
Bipolar bone loss resulting in an “off-track” lesion	Glenoid bone loss exceeding that which can be addressed with the coracoid

**Table 2:** Preoperative imagistic landmarks.

Preoperative Imagistic Landmarks
Hill–Sachs interval—HSI (cm) (the distance from the rotator cuff attachments to the medial rim of the Hill–Sachs lesion) (Figure 2)
Glenoid track—GT(0.83 × D-d) − D = the estimated diameter of the intact glenoid (cm), d = the width of the anterior glenoid bone defect (cm) (Figure 4)
HSI/GT (cm/cm): on-track lesion: HSI < GT : off-track lesion: HSI > GT (imagistic criteria for Latarjet surgery)
Glenoid bone loss (%)—GBL (Figure 4)
Glenoid index (cm/cm)—GI (height-to-width ratio) (Figure 4)
Retroversion or anteversion of glenoid cavity

**Table 3:** The rehabilitation phases.

The Rehabilitation Phases
Phase 1: Passive mobilisation for restoring ROM and scapular stabilisation (ER: 20°, IR: 90°, Abduction: 80–90°, Flexion: 100–120°)
Phase 2: Active-assisted mobilisation (at 6 weeks postoperatively)
Phase 3: Active mobilisation with progressive muscle strengthening.

**Table 4:** Clinical and imaging characteristics of the cohort.

Characteristic	Result
Number of patients	16
Men, n (%)	12 (75.00%)
Age, years	25 (20–42)
Right shoulder operated, n (%)	9 (56.25%)
Dominant shoulder operated, n (%)	7 (43.75%)
Preoperative dislocations	9.50 (7.50–15.00)
Glenoid bone loss, %	22.19 ± 5.17
Hill–Sachs interval (cm)	2.77 (2.64–2.83)
Glenoid track (cm)	2.00 ± 0.22
HSI/GT ratio (cm/cm)	1.41 ± 0.23
Glenoid index (cm/cm)	1.77 ± 0.18
Glenoid version °	7.05 (6.78–7.90)

**Table 5:** Functional outcomes and residual symptoms.

**Outcome**		**Value**
Recovery time, months		4 (3.00–6.00)
Flexion, °		180 (180.00–180.00)
External rotation, °		45 (45.00–90.00)
Internal rotation, n (%)	Between the scapula	3 (18.75%)
	Below the scapula	11 (68.75%)
	Lumbar level	2 (12.5%)
Rowe score		95.00 (90.00–95.00)
Walch–Duplay score		75.00 (70.00–90.00)
Return to same level, n (%)		7 (43.75%)
Lower level, n (%)		4 (25.00%)
Changed sport, n (%)		4 (25.00%)
Stopped activity, n (%)		1 (6.25%)
Residual limitation, n (%)		6 (37.50%)
Pain during activity, n (%)		5 (31.25%)
Sensation of instability, n (%)		2 (12.50%)

**Table 6:** All radiologic–clinical associations tested. (Appendix A).

Predictor	Outcome Score	ρ	95% CI	*p*	FDR q
HSI/GT	Walch–Duplay	−0.64	[−0.89; −0.20]	0.007	0.043
HSI/GT	Rowe	−0.59	[−0.88; −0.13]	0.014	0.043
GBL(%)	Walch–Duplay	−0.21	[−0.70; 0.28]	0.420	0.420
GBL (%)	Rowe	−0.59	[−0.80; −0.17]	0.015	0.043
GI	Walch–Duplay	−0.58	[−0.87; −0.10]	0.017	0.043
GI	Rowe	−0.32	[−0.77; 0.24]	0.228	0.253
GT	Walch–Duplay	0.52	[0.06; 0.82]	0.036	0.072
GT	Rowe	0.37	[−0.10; 0.75]	0.148	0.211
HSI	Walch–Duplay	−0.38	[−0.77; 0.21]	0.145	0.211
HSI	Rowe	−0.34	[−0.76; 0.27]	0.190	0.237

**Table 7:** Exploratory comparison according to postoperative level of activities.

Variable	Same level (n = 7)	Other Outcome (n = 9)	*p*	FDR q
Recovery time (months)	3.00 [2.50; 4.50]	4.00 [4.00; 6.00]	0.174	0.261
External rotation (°)	90 [45; 90]	45 [45; 45]	0.142	0.261
Rowe score	95.00 [92.50; 95.00]	90.00 [80.00; 95.00]	0.252	0.302
HSI/GT ratio	1.27 ± 0.13	1.52 ± 0.23	0.021	0.126
GBL (%)	20.00 [18.50; 21.00]	23.00 [19.00; 27.00]	0.536	0.536
GI	1.68 ± 0.11	1.84 ± 0.19	0.059	0.177

**Figure A1:**
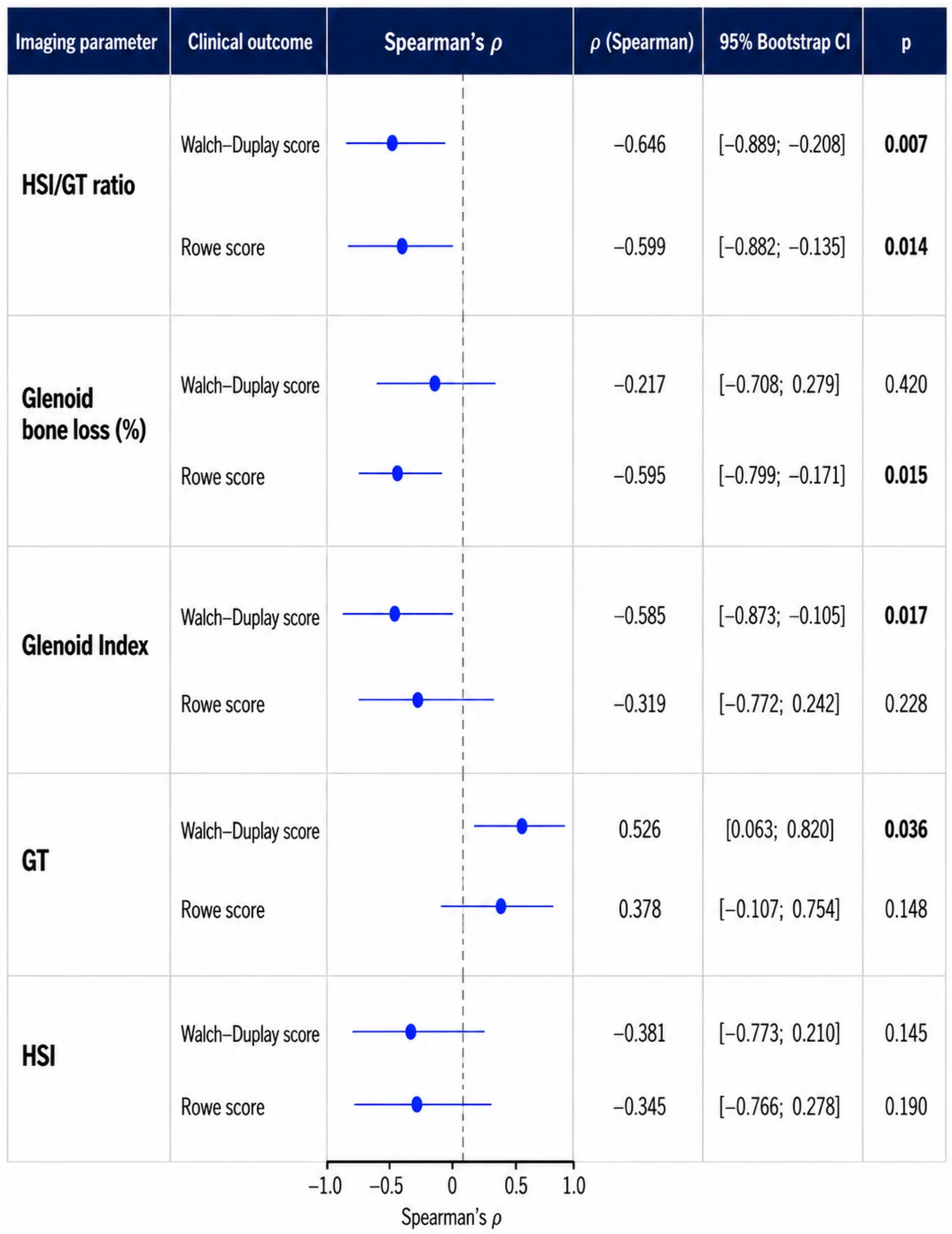
Correlation between preoperative imaging landmarks and 6-month follow-up outcomes.

## Data Availability

The data presented in this study are available on request from the corresponding author.

## Abbreviations

CT	Computed tomography
MRI	Magnetic resonance imaging
CP	Coracoid process
GBL	Glenoid bone loss
HSI	Hill–Sachs interval
GT	Glenoid track
GI	Glenoid index
